# The Cutting Edge: A Systematic Review of Artificial Intelligence and Machine Learning in Predicting Esophagectomy Outcomes

**DOI:** 10.1016/j.atssr.2026.02.002

**Published:** 2026-02-18

**Authors:** Divyaam Satija, Ahmed Aly, Theodore Dimitrov, Dev Mookerjee, Jeremy Chang, Junyi Liu, Robert E. Merritt, Yuan Xue, Peter J. Kneuertz

**Affiliations:** 1 Division of Thoracic Surgery, Department of Surgery, The Ohio State University, Columbus, Ohio; 2 Department of Biomedical Informatics, The Ohio State University, Columbus, Ohio

## Abstract

**Background:**

Esophagectomy carries high risks and complications. Traditional outcome prediction models often fail to account for the multifactorial nature of postoperative outcomes. This systematic review evaluates the use of artificial intelligence and machine learning (AI/ML) models in predicting outcomes after esophagectomy.

**Methods:**

A systematic review of PubMed and Embase was conducted to identify studies published from January 2014 to June 2024. Inclusion criteria were observational studies or clinical trials using AI/ML models to predict esophagectomy outcomes. Studies were assessed for quality, and data were extracted on study characteristics, patient demographics, AI/ML models used, and predictive outcomes.

**Results:**

Of 187 studies identified, 19 met the inclusion criteria. These studies, conducted across various countries, employed models such as random forest, neural networks, and gradient boosting machines. The most common outcomes predicted were postoperative complications, long-term survival, and early recurrence and readmission. Random forest models frequently demonstrated strong predictive performance, with area under the curve values often exceeding 0.8. The studies demonstrated the potential of AI/ML in enhancing prediction accuracy but highlighted significant variability in models and methodologies.

**Conclusions:**

AI/ML models show promise in enhancing the predictive accuracy of esophagectomy outcomes. However, the variability in methodologies underscores the need for standardization in model development and validation. Future research should focus on larger, multi-institutional studies to improve the generalizability and clinical applicability of these models.

**In Short**

▪ Artificial intelligence and machine learning models show promise in predicting postesophagectomy outcomes such as complications, survival, and recurrence, often outperforming traditional methods.
▪ Significant variability in models, data inputs, and outcome measures still limit comparability and clinical adoption.
▪ Standardization, multi-institutional validation, and attention to ethical factors are essential for integrating artificial intelligence and machine learning into surgical decision-making.

Esophageal cancer remains a significant challenge in thoracic surgery because of its high morbidity and mortality rates.^1^ Esophagectomy, the surgical removal of a portion of the esophagus and stomach, is commonly performed to treat early-stage esophageal cancer. Despite curative intent, it is one of the most complex thoracic surgeries, with considerable risks that can affect recovery, long-term health, and quality of life.^2^^,^^3^ Complications such as pneumonia, anastomotic leak, chyle leak, and diaphragm impairment are potentially life-threatening and contribute to significant patient morbidity and prolonged recovery. Traditional methods for assessing and predicting outcomes in esophageal cancer currently rely on clinical parameters and scoring systems but often fail to account for the multifactorial nature of patient risks. Studies have reported that 20% to 40% of esophagectomy patients experience complications, with respiratory failure being a leading cause of postoperative mortality.^2^^,^^3^ Nonetheless, current models do not adequately integrate the complex cardiopulmonary histories, fitness, and nutrition status of patients, resulting in suboptimal prediction accuracy.

Given these limitations, recent years have seen the emergence of artificial intelligence (AI) and machine learning (ML) technologies that demonstrate significant potential in health care, particularly in improving diagnostic accuracy, treatment planning, and outcome prediction. These technologies excel in analyzing large data sets and identifying intricate patterns that traditional methods may overlook. In oncology, AI/ML has been successfully applied to predict outcomes across multiple cancer types.^4^ The promising results from these applications suggest that AI/ML could also enhance predictive accuracy for esophagectomy outcomes. Despite the potential benefits, the application of AI/ML in esophagectomy outcomes is still in its nascent stages. Although initial studies showed improved predictions, they were limited by small sample sizes, heterogeneous methodologies, and a lack of standardized evaluation metrics. There is a clear need for systematic investigation to validate the effectiveness of AI/ML in this context.

This systematic review aims to evaluate the current evidence on the use of AI/ML in predicting esophagectomy outcomes. By synthesizing existing research, we seek to identify the strengths and limitations of these models and to provide recommendations for future studies to advance this promising field.

## Material And Methods

### Literature Search

This systematic review was conducted and reported in accordance with Preferred Reporting Items for Systematic Reviews and Meta-Analyses 2020 guidelines. An electronic search was conducted in PubMed and Embase to identify studies evaluating the impact of AI/ML on esophagectomy outcomes. References were screened to remove duplicates and to ensure comprehensive coverage. The search strategy was developed with use of a combination of Medical Subject Headings (MeSH) terms and free-text keywords related to AI, ML, esophageal cancer, and esophagectomy. The search was limited to studies published in English from January 2014 to June 2024. The final search was performed on June 1, 2025. The complete search strategy, including MeSH terms and free-text keywords for each database, is provided in the Supplemental Table. Institutional review board approval was not required for this study.

### Inclusion and Exclusion Criteria and Data Extraction

Studies were considered eligible for inclusion if they met the following criteria: observational, clinical, or cohort studies; included patients undergoing surgical treatment of esophageal cancer; and implemented AI/ML to predict or to analyze outcomes. Studies were excluded if they involved non-human subjects. Titles and abstracts were screened by our reviewer (D.S.), with full-text review and final inclusion confirmed in discussion with the senior author (P.K.). Data extractions were performed with a standardized data extraction form to obtain the following: study characteristics, including authors, publication year, country, study design, sample size, and duration of follow-up; patient characteristics, including age, sex, stage of cancer, and type of procedure; type of AI/ML model used, input variables, model training and validation processes, and outcomes; and surgical outcomes and other relevant postoperative metrics.

The Downs and Black quality assessment tool was employed to assess the methodologic quality and risk of bias of nonrandomized studies. This validated instrument consists of 27 items evaluating reporting quality and internal and external validity. Given the observational nature of the included studies, the power assessment item (question 27) was excluded. The maximum possible score was therefore 26. Studies scoring 0 to 9 were classified as low quality; 10 to 18, moderate quality; and 19 to 26, high quality.

## Results

### Study Selection and Population Distribution

**Figure 1:**
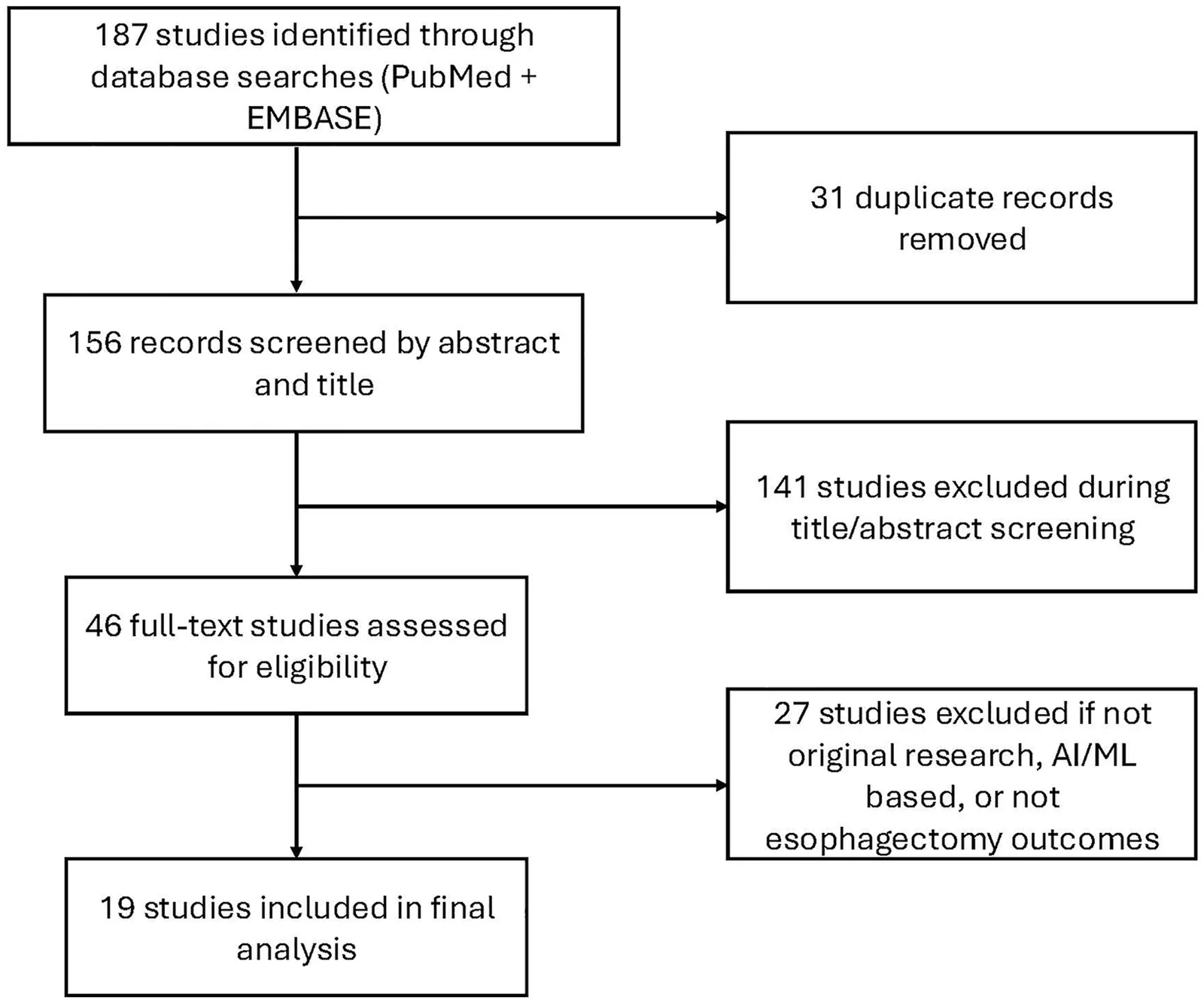
Preferred Reporting Items for Systematic Reviews and Meta-Analyses flow diagram of study selection process. (AI/ML, artificial intelligence and machine learning.)

**Figure 2:**
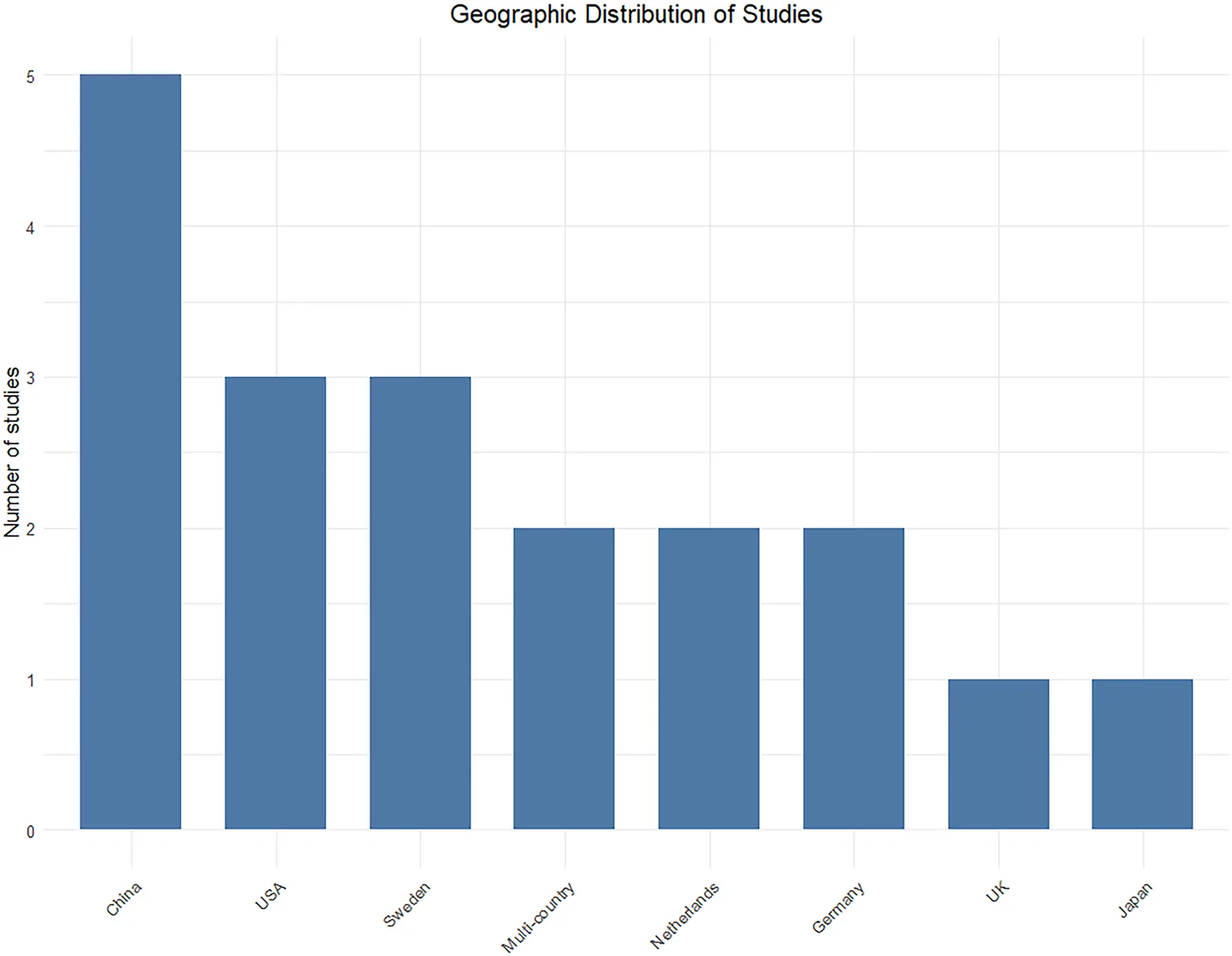
Geographic distribution of studies. (UK, United Kingdom; USA, United States.)

A total of 187 studies were initially identified, with 19 studies finally included (Figure 1). On the basis of the Downs and Black assessment, all studies were of moderate or higher methodologic quality, with common limitations related to retrospective design and limited external validation. These studies were conducted across several countries including the United States, the United Kingdom, China, and others (Figure 2).^5^, ^6^, ^7^, ^8^, ^9^, ^10^, ^11^, ^12^, ^13^, ^14^, ^15^, ^16^, ^17^, ^18^, ^19^, ^20^, ^21^, ^22^, ^23^ Sample sizes ranged from 60 to 6399 patients, with median ages typically ranging from 60 to 70 years (Table 1).

**Table 1:** Study Characteristics

Study	Study Type	Region	No. of Patients	Patient Population
Rahman, 2020	Retrospective	United Kingdom and The Netherlands	812	Adenocarcinoma
Tang, 2021	Prospective	United States	77	Mixed
Bolourani, 2021	Retrospective	United States	2037	Mixed
Wang, 2021	Retrospective	China	153	Squamous
Zhao, 2021	Retrospective	China	710	Mixed
Kouzu, 2022	Retrospective	Japan	222	Squamous
Hu, 2022	Retrospective	China	533	Squamous
van Kooten, 2022	Retrospective	The Netherlands	4228	Mixed
Lyu, 2023	Retrospective	United States	260	Mixed
Tong, 2023	Retrospective	China	155	Squamous
Rahman, 2023	Retrospective	United Kingdom	6399	Mixed
Jung, 2023	Retrospective	Germany	1360	Adenocarcinoma
Gujjuri, 2023	Retrospective	Europe	4719	Mixed
Åkesson, 2023	Prospective	Sweden	60	Mixed
Jung, 2023	Retrospective	Germany	864	Mixed
Li, 2023	Retrospective	China	194	Squamous
Klontzas, 2024	Prospective	Sweden	471	Mixed
van de Beld, 2024	Retrospective	The Netherlands	417	Mixed
Djerf, 2024	Retrospective	Sweden	1846	Mixed

### AI Models and Techniques Used

**Figure 3:**
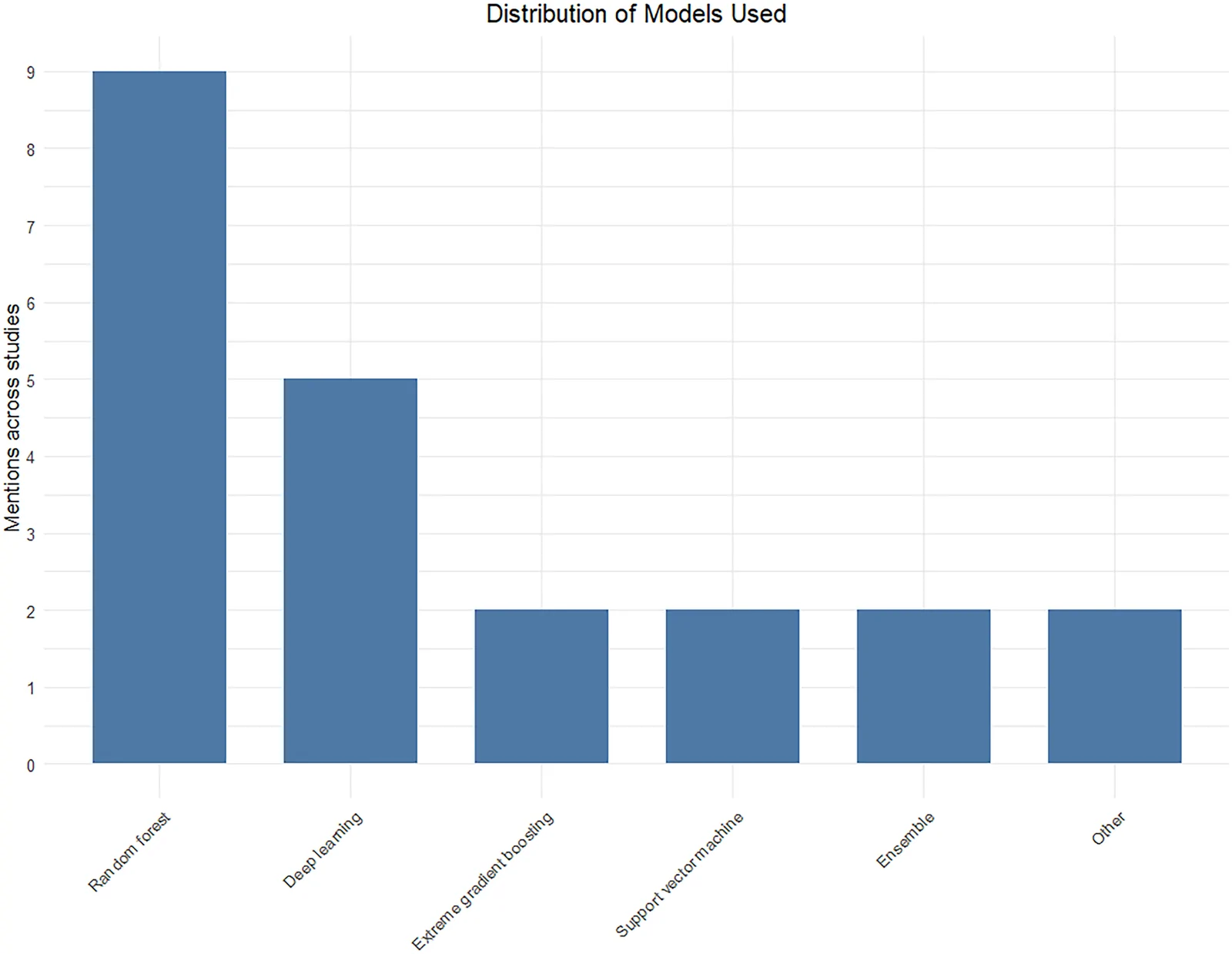
Distribution of artificial intelligence and machine learning models used.

In the studies analyzed, random forest was the most frequently employed model, appearing in 9 of the 19 studies (Figure 3; Table 2). It demonstrated strong performance in predicting various outcomes, such as in the study by Bolourani and coworkers,^7^ in which it achieved an area under the curve (AUC) of 0.74 for predicting early readmission. Deep learning models, including neural networks, were used in 5 studies, showing high accuracy in complex data sets. For example, Jung and coworkers^19^ used it to predict severe postoperative complications, consistently outperforming logistic regressions.

**Table 2:** Features, Outcomes, and Performance

Study	AI Model	Preoperative vs Postoperative	Validation	Outcomes	Performance Metrics (AUC)
Rahman, 2020	Ensemble model	Preoperative/postoperative	Bootstrap	Early recurrence	0.81
Tang, 2021	RF (imbalanced)	Preoperative	Out-of-bag	30-day morbidity	G-mean error 0.32
Bolourani, 2021	RF	Preoperative, intraoperative, postoperative	4-fold CV	30-day readmission	0.74
Wang, 2021	RSF + deep survival	Preoperative/postoperative	5-fold CV	Overall survival	C index 0.69
Zhao, 2021	LASSO + RF + naive Bayes	Preoperative, intraoperative, postoperative	90/10 split + 9-fold CV	Anastomotic leakage	0.72
Kouzu, 2022	Neural network	Postoperative	Train/test split	Disease-specific survival	0.81
Hu, 2022	LASSO-Cox nomogram	Preoperative	70/30 split	Overall survival	C index > TNM-staging
van Kooten, 2022	Ensemble	Preoperative/intraoperative	75/25 split + CV	Leak and pulmonary complications	0.62-0.64
Lyu, 2023	RF + naive Bayes	Preoperative/postoperative	5-fold CV	Anytime recurrence	0.82
Tong, 2023	Logistic nomogram	Preoperative	Multiple	4-year recurrence	0.83-0.85
Rahman, 2023	RSF	Preoperative, intraoperative, postoperative	Bootstrap	5-year overall survival	0.78
Jung, 2023	RSF	Preoperative/postoperative	Halving grid-search CV	Overall survival	C index 0.74
Gujjuri, 2023	RSF	Preoperative/postoperative	Bootstrap	5-year overall and disease-free survival	0.78
Åkesson, 2023	Neural networks	Postoperative	70/30 split	Anastomotic defect	1.0
Jung, 2023	RF + neural network	Preoperative	3-fold CV	Major complications	0.67
Li, 2023	Radiomics nomogram	Preoperative	3-fold CV	Pathologic complete response	0.82-0.90
Klontzas, 2024	XGBoost	Preoperative	10-fold CV	Anastomotic leakage	0.79
van de Beld, 2024	Temporal deep learning	Preoperative/postoperative	5-fold CV	Leakage 1 day ahead	0.87
Djerf, 2024	XGBoost	Preoperative/intraoperative	90/10 split + CV	90-day mortality and leak	0.91/0.84

AI, artificial intelligence; AUC, area under the curve; CV, cross-validation; G-mean, geometric mean; LASSO, least absolute shrinkage and selection operator; RF, random forest; RSF, random survival forest; XGBoost, extreme gradient boosting.

Gradient boosting machines and support vector machines were each employed in 2 studies, for nonlinear data. Elastic net regression models were less common, used in 1 study by Rahman and coworkers^15^ as part of an ensemble approach, which delivered the highest performance for early recurrence prediction.

Validation techniques included k-fold cross-validation, ensuring that models were tested on multiple data subsets. Bootstrapping was also used in some studies to enhance the robustness of estimates, particularly in smaller sample sizes. Finally, studies with larger data sets often used split data sets for training and testing, ensuring thorough evaluation of model performance (Table 2). AUC values were the primary metric reported for model performance, with several studies achieving AUCs above 0.8 (Table 2).

### Predictive Outcomes Studied

#### Postoperative Complications

The studies examined a wide array of clinical outcomes ranging from early recurrence and readmission to overall survival and postoperative complications. The most common were postoperative complications. Tang and colleagues^6^ introduced a new frailty index, the Esophageal Vitality Index, to successfully predict postoperative outcomes using simple physiologic metrics. Jung and coworkers,^19^ in contrast, employed neural networks using clinical variables to predict severe complications. Their neural networks model successfully outperformed logistic regressions in predictions every time. Van de Beld and coworkers^22^ used temporal models incorporating laboratory results and vital signs, which improved prediction of anastomotic leakage and pneumonia. Similarly, Bolourani and coworkers^7^ used a random forest with a NearMiss algorithm to predict postoperative outcomes. Finally, Klontzas and coworkers^21^ as well as Djerf and coworkers^23^ reported that extreme gradient boosting (XGBoost) models using computed tomography (CT) imaging data and clinical data had high AUCs in predicting postoperative outcomes.

#### Long-Term Survival

Long-term survival was the second most common outcome studied by the papers, with 6 studies evaluating survival in this population. For instance, Wang and colleagues^8^ combined CT radiomics and histopathologic features, with a combined model achieving a C index of 0.694. Rahman and coworkers^15^ and Gujjuri and coworkers,^17^ in contrast, used random survival forest models, achieving a 5-year AUC of 83.9%, outperforming Cox regression models from the same data sets. Finally, Hu and colleagues^11^ developed a model integrating clinical data as well as biomarker data, improving accuracy over traditional features.

#### Recurrence and Readmission

Four studies targeted recurrence and readmission predictions. Rahman and coworkers^5^ developed an ensemble model with an AUC of 0.81, identifying lymphovascular invasion as a key predictor. Lyu and coworkers^13^ and Bolourani and coworkers,^7^ in contrast, used various machine learning models, with random forest showing a consistently higher performance over all others. Finally, Tang and colleagues^6^ developed a radiomics-based nomogram that combined clinical variables and radiomic features, achieving AUCs of 0.85 and 0.83 in different cohorts.

## Comment

In recent years, AI/ML models have garnered considerable interest in their potential to enhance predictive accuracy in esophagectomy outcomes. Existing literature in other oncologic fields has demonstrated the efficacy of AI/ML models in improving predictive outcomes and optimizing treatment strategies. However, our systematic review reveals that whereas AI/ML models hold promise in the context of esophagectomy, the current body of evidence remains limited and fragmented, reflecting the nascent stage of research in this area. AI/ML models have the potential to transform traditional predictive methodologies, which often fail to fully capture the multifactorial drivers of postoperative outcomes.

It is evident from this review that AI/ML models have shown considerable potential in predicting a range of postoperative outcomes, including complications, long-term survival, and early recurrence and readmission. Random forest models were frequently used because of their strong performance in handling complex, nonlinear data, whereas neural networks consistently outperformed traditional models, highlighting their efficacy in managing intricate data sets. In addition, models such as that developed by Wang and colleagues^8^ demonstrated that integrating CT-based radiomic features with histopathologic data improved survival prediction compared with clinical variables alone, underscoring the potential value of multimodal AI/ML approaches. Collectively, these findings suggest that AI/ML methods may capture complex interactions between patient, procedural, and perioperative factors better than conventional risk prediction tools.

Despite these encouraging findings, the significant variability in AI/ML algorithms, data sources, and outcome measures across the reviewed studies presents a major challenge to the field. This variability complicates the comparison of results and diminishes the generalizability of individual findings. In addition, as these technologies become more integrated into surgical practice, ethical and regulatory challenges will become increasingly significant. Issues such as algorithmic bias, data privacy, and the robustness of AI/ML models in diverse clinical settings must be addressed to ensure safe and equitable implementation.^24^

To advance the field and to address these concerns, it is crucial to establish standardized approaches to AI/ML model development, training, and validation. For instance, postoperative complications should be defined by established severity-based frameworks with prespecified horizons (30- vs 90-day outcomes), whereas survival outcomes should clearly distinguish overall and disease-specific survival at prespecified time horizons (5-year vs 7-year survival). Model performance should be reported with both discrimination metrics (AUC vs C index) and calibration measures (calibration slope vs Brier score). Finally, validation strategies should also be explicitly reported and standardized, including clear specification of internal validation methods (k-fold validation vs bootstrapping). Adoption of these specific standards would substantially improve comparability and clinical interpretability across studies.^23^ With ongoing research and multi-institutional collaborations, AI/ML could truly play a transformative role in esophagectomy.

### Limitations

Although our findings contribute valuable insights, it is important to consider limitations that may have an impact on their interpretation. The most notable limitation is the significant heterogeneity in the AI/ML models, input variables, and outcomes across the included studies, which complicates direct comparisons and synthesis of findings. The lack of standardized evaluation metrics further exacerbates this challenge, making it difficult to draw definitive conclusions about the relative effectiveness of different AI/ML approaches. In addition, many studies included in this review were retrospective and conducted at single institutions with relatively small sample sizes, which may limit generalizability of the results. Another limitation is the exclusion of studies from non-English language publications, which might have omitted relevant data from other regions that could have provided a more global perspective. Moreover, it is also important to acknowledge the potential publication bias toward positive findings in AI/ML research, which may overstate the models’ efficacy. Lastly, the rapidly evolving nature of AI/ML technologies means that some studies may have used earlier, less sophisticated versions of the models that do not fully reflect the current capabilities of AI/ML in esophagectomy.

### Conclusion

This systematic review summarizes the current evidence and highlights future potential of AI/ML to enhance predictive accuracy in esophagectomy, offering opportunities for more personalized patient care. However, ethical challenges as well as significant variability in models and outcomes highlight the need for standardized frameworks for AI/ML development and validation. Future research should focus on multi-institutional collaborations and large, diverse populations to improve the generalizability and reliability of AI/ML models. As the field advances, AI/ML could play a crucial role in improving surgical outcomes and patient quality of life after esophagectomy.
