# Supplementary material for: The Cutting Edge: A Systematic Review of Artificial Intelligence and Machine Learning in Predicting Esophagectomy Outcomes
Source: Ann Thorac Surg Short Rep. 2026 Feb 18;4(3):1015–22. doi: 10.1016/j.atssr.2026.02.002 (PMC13555475; doi:10.1016/j.atssr.2026.02.002)
Supplement: Supplementary file 1 [file mmc1.docx]

**Supplementary Table 1.** Search terms used with for Boolean logic: *(Disease terms) AND (Procedure terms) AND (Artificial intelligence / machine learning terms)*

| PubMed | | |
| --- | --- | --- |
| Disease terms | Procedure terms | Artificial intelligence / machine learning terms |
| "Esophageal Neoplasms"[MeSH] esophageal cancer oesophageal cancer | "Esophagectomy"[MeSH] esophagectomy | - "Artificial Intelligence"[MeSH] - "Machine Learning"[MeSH] - artificial intelligence - machine learning - deep learning - neural network* - random forest* |
| Embase | | |
| Disease terms | Procedure terms | Artificial intelligence / machine learning terms |
| 'esophageal cancer'/exp esophageal cancer oesophageal cancer | 'esophagectomy'/exp esophagectomy | - 'artificial intelligence'/exp - 'machine learning'/exp - artificial intelligence - machine learning - deep learning - neural network* |
